# Position 123 of halohydrin dehalogenase HheG plays an important role in stability, activity, and enantioselectivity

**DOI:** 10.1038/s41598-019-41498-2

**Published:** 2019-03-25

**Authors:** Jennifer Solarczek, Thomas Klünemann, Felix Brandt, Patrick Schrepfer, Mario Wolter, Christoph R. Jacob, Wulf Blankenfeldt, Anett Schallmey

**Affiliations:** 1 Institute for Biochemistry, Biotechnology and Bioinformatics, Technische Universität Braunschweig, Spielmannstr. 7, 38106 Braunschweig, Germany; 2 Structure and Function of Proteins, Helmholtz Centre for Infection Research, Inhoffenstr. 7, 38124 Braunschweig, Germany; 3 Institute of Physical and Theoretical Chemistry, Technische Universität Braunschweig, Gaußstr. 17, 38106 Braunschweig, Germany

## Abstract

HheG from *Ilumatobacter coccineus* is a halohydrin dehalogenase with synthetically useful activity in the ring opening of cyclic epoxides with various small anionic nucleophiles. This enzyme provides access to chiral β-substituted alcohols that serve as building blocks in the pharmaceutical industry. Wild-type HheG suffers from low thermostability, which poses a significant drawback for potential applications. In an attempt to thermostabilize HheG by protein engineering, several single mutants at position 123 were identified which displayed up to 14 °C increased apparent melting temperatures and up to three-fold higher activity. Aromatic amino acids at position 123 resulted even in a slightly higher enantioselectivity. Crystal structures of variants T123W and T123G revealed a flexible loop opposite to amino acid 123. In variant T123G, this loop adopted two different positions resulting in an open or partially closed active site. Classical molecular dynamics simulations confirmed a high mobility of this loop. Moreover, in variant T123G this loop adopted a position much closer to residue 123 resulting in denser packing and increased buried surface area. Our results indicate an important role for position 123 in HheG and give first structural and mechanistic insight into the thermostabilizing effect of mutations T123W and T123G.

## Introduction

Halohydrin dehalogenases (HHDHs; also, haloalcohol dehalogenases or epoxidases, halohydrin hydrogen-halide-lyases) are biotechnologically relevant enzymes for the production of chiral epoxides and β-substituted alcohols^1^. In their natural function, they catalyze the reversible dehalogenation of vicinal haloalcohols with formation of the corresponding epoxides. In the reverse reaction, i.e. epoxide ring opening, these enzymes do not only accept halides but also other anionic nucleophiles such as azide, cyanide, nitrite, cyanate or formate^2^. This enables the formation of novel carbon-carbon, carbon-nitrogen or carbon-oxygen bonds by HHDH catalysis. Currently, their most important biocatalytic application is the synthesis of side chain precursors for cholesterol-lowering drugs (statins) such as atorvastatin and rosuvastatin^3–6^.

HHDHs are members of the short-chain dehydrogenases/reductases (SDR) superfamily^7,8^. Thus, they share several structural as well as mechanistic features with SDR enzymes albeit catalyzing chemically different reactions. HHDHs possess a catalytic triad composed of serine, tyrosine and arginine. Moreover, they are cofactor-independent. Hence, the nicotinamide cofactor-binding pocket found in SDR enzymes is replaced by a nucleophile-binding pocket in HHDHs.

Unlike SDR enzymes, halohydrin dehalogenases are relatively rare in nature and were only found in bacteria so far. Until 2013, the gene sequences of merely a handful of different HHDH enzymes – *hheA* from *Corynebacterium* sp. strain N-1074^9^, *hheA2* from *Arthrobacter* sp. strain AD2^7^, *hheB* from *Corynebacterium* sp. strain N-1074^9^, *hheB2* from *Mycobacterium* sp. strain GP1^7,10^, and *hheC* identified in *Agrobacterium tumefaciens* AD1^7^ and *Rhizobium* sp. strain NHG3^11^ – were known. These have been classified into three different enzyme subtypes A, B and C based on sequence homology and substrate specificities^7^. Crystal structures of HheA^12^, HheA2^13^, HheB^12^ and HheC^14^ have been determined and especially HheC and HheA2 have been engineered extensively already^1^.

Since the identification of HHDH-specific sequence motifs^15^, a large number of novel HHDH sequences has been discovered in public sequence databases. This way, the phylogenetic classification of HHDHs could be expanded by subtypes D through G. During detailed characterization of a representative set of 17 new HHDH enzymes^16^, HheG from *Ilumatobacter coccinues* displayed high activity in the conversion of cyclohexene oxide and limonene oxide^17^. Thus, this enzyme is the first reported halohydrin dehalogenase with synthetically useful activity on cyclic epoxides. Determination of the crystal structure of HheG revealed the presence of a wide, solvent-exposed cleft harboring the active site^17^. This is in contrast to more buried active site pockets of other HHDH structures and likely explains the exceptional substrate scope of this enzyme. HheG even displayed moderate enantioselectivity in the ring opening of cyclohexene oxide with azide and cyanide as nucleophiles. Stereoselective ring opening of cyclic epoxides offers access to a range of cyclic β-substituted alcohols that are building blocks of active pharmaceutical ingredients^18,19^. Therefore, HheG is a promising biocatalyst for future industrial application.

During our biochemical characterization, however, a very low thermostability was observed for HheG, as the enzyme was inactive at temperatures ≥40 °C. This poses a significant drawback for potential application of this HHDH on large scale as low thermostability is usually accompanied by low process stability^20^. Therefore, we aimed to thermostabilize HheG by means of protein engineering. Thermostabilization of HHDHs, especially HheC from *A. tumefaciens* AD1, has been attempted previously using either random mutagenesis or computational approaches to identify hot spots, and subsequent combination of beneficial mutations to increase thermostability further^21,22^. Computational approaches in protein engineering, compared to random mutagenesis, can help to significantly reduce the number of variants that have to be screened if knowledge on the enzyme structure is available. As the latter is available for HheG, we opted for a semi-rational engineering approach to generate thermostable HheG variants.

## Results and Discussion

### Thermostabilization of HheG

**Figure 1:**
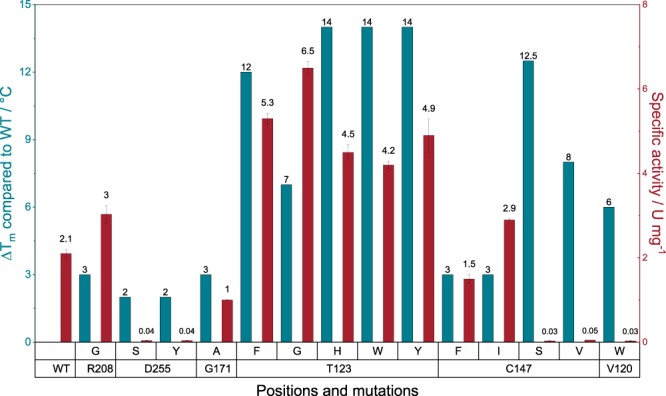
Melting temperature deviations (ΔT_m_) compared to wild-type HheG (WT) and specific activities of positive hits from the thermostability screening. Melting temperatures were determined via thermofluor assay^24^ and specific activities were measured for epoxide ring opening of cyclohexene oxide with azide as nucleophile.

Based on the crystal structure of HheG (PDB: 5O30^17^) and on different computational approaches, a total of twenty amino acid residues were selected for protein engineering of HheG to improve its thermostability. The selection was based on (i) the flexibility of amino acids (B-factor values), (ii) the possibility to introduce new hydrogen bonds in flexible loops and (iii) the introduction of additional interactions between subunits, (iv) the increase of surface hydrophilicity and (v) predictions from the FireProt web server^23^. Selected amino acid positions were either randomized to yield site saturation mutagenesis libraries, or exchanged by specific amino acids yielding point mutants. The apparent melting temperature (T_m_) of all variants was determined using the thermofluor assay^24^ and compared to wild-type HheG, which displayed a T_m_ of 38 °C. In total, 865 variants were screened, and 14 of these exhibited an increased apparent melting temperature of at least +2 °C (Fig. 1). Interestingly, two amino acid positions, T123 and C147, could be identified that yielded single mutants with a ΔT_m_ > +10 °C. Both residues were predicted by the FireProt web server^23^ to affect the enzyme’s thermostability. While residue T123 of HheG sits in a long helix that forms part of a four-helix bundle in the dimer interface, residue C147 is positioned in the central β-sheet of the monomer.

**Figure 2:**
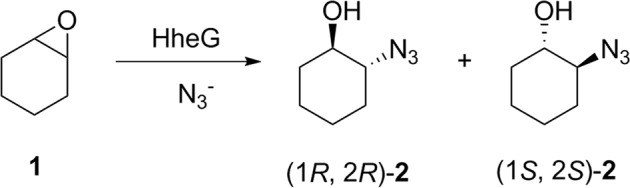
Epoxide ring-opening reaction of cyclohexene oxide (**1**) with HheG and azide yielding 2-azido-cyclohexan-1-ol (**2**).

As we were aiming for more thermostable HheG variants with uncompromised activity, specific activities of all 14 thermostable variants in the epoxide ring-opening of cyclohexene oxide were determined using azide as nucleophile (Fig. 2) and compared to HheG wild-type (Fig. 1). Interestingly, most thermostabilizing mutations had a significant effect on enzyme activity as well. Gratifyingly, amino acid exchanges T123G, T123F, T123Y, T123W and T123H did not only yield variants with 7 to 14 °C higher T_m_, but also up to 3-fold increased specific activity. The latter was unexpected, as residue T123 does not form part of the enzyme active site. In contrast, thermostabilizing mutations at position C147 either resulted in a dramatic drop in enzyme activity by two orders of magnitude or altered enzyme activity only slightly (Fig. 1).

It should be noted here that also HheG variants with unchanged (e.g. T123I and T123L) or significantly decreased apparent melting temperature (e.g. T123N and T123P with ΔT_m_ of −9 and −10 °C, respectively) were obtained for position 123. Of these, variant T123I also displayed only very low residual activity (data not shown). Hence, amino acid position 123 seems to play a special role in HheG regarding activity and stability of the enzyme.

Beneficial mutations of variants with increased T_m_ and increased specific activity were combined to further improve HheG’s thermostability. However, all resulting double mutants displayed lower T_m_ values compared to our best single mutants (see Supplementary Table S2), and were therefore not investigated further. This is in contrast to the thermostabilization of HheC, for which a further improvement in T_m_ could be achieved by almost all combinations of beneficial mutations^21^.

### Characterization of HheG T123 single mutants

First, apparent melting temperatures of HheG wild-type and variants T123F, T123G and T123W were also determined by CD spectroscopy, which confirmed the significant impact of the respective mutations on the enzyme’s thermostability (Table S3).

For a detailed comparison of variants T123G, T123F, T123Y, T123W and T123H with HheG wild-type regarding catalytic efficiency, determination of enzyme kinetic parameters in the ring-opening reaction of cyclohexene oxide with azide was attempted. This, however, was hampered by low binding affinities of all enzymes for the substrate and the low solubility of cyclohexene oxide in the reaction medium (0.5 g L^−1^ in water, according to the manufacturer). Hence, addition of a significant amount of co-solvent was required, which in turn affected the activity of all tested enzymes negatively (see also Supplementary Fig. S2). Therefore, initial reaction velocities for wild-type HheG and T123 single mutants were determined instead, using a fixed substrate and co-solvent concentration. Reactions were performed at a pH of 7, since the pH optimum of wild-type HheG in the azidolysis of cyclohexene oxide was previously shown to be around pH 6 to 7^17^. As a result, all T123 single mutants displayed significantly higher initial reaction rates compared to wild-type (Table 1). Among the aromatic mutants T123W, T123Y and T123H, which displayed the highest increase in apparent melting temperature (ΔT_m_ = +14 °C), T123H was the most active one with a 3.3-fold higher initial reaction velocity. Moreover, variant T123G with a ΔT_m_ of +7 °C exhibited the highest increase in activity with a 5-fold higher initial rate compared to wild-type.

**Table 1:** Initial reaction velocity and product enantiomeric excess (ee_P_) for the epoxide ring opening of cyclohexene oxide with azide using HheG wild-type (WT) and T123 mutants.

	Initial reaction velocity/µmol min^−1^	Enantiomeric excess ee_P_/%
HheG WT pH 8	0.058 ± 0.001	40^17^
HheG WT pH 7	0.106 ± 0.003	52
HheG T123W	0.184 ± 0.001	64
HheG T123H	0.350 ± 0.007	63
HheGT123Y	0.338 ± 0.004	63
HheG T123F	0.442 ± 0.011	64
HheG T123G	0.523 ± 0.028	55

To compare the stereoselectivity of the T123 single mutants with wild-type HheG, the enantiomeric excesses (ee_P_) of the formed azidoalcohol products in the azidolysis of cyclohexene oxide were determined. For HheG wild-type, an ee_P_ of 40% with preferential formation of the (1*S*, 2*S*)-enantiomer (Scheme 1) has previously been obtained at pH 8^17^. When we now repeated the reaction at pH 7, the resulting product enantiomeric excess was already slightly increased (ee_P_ = 52%, Table 1). This is likely due to the observed higher activity of wild-type HheG in the azidolysis of cyclohexene oxide at pH 7 compared to pH 8, and thus an overall lower negative effect of the chemical (i.e. not enzyme-catalyzed) background reaction on the final product enantiomeric excess. Interestingly, exchange of T123 by an aromatic amino acid, as present in variants T123W, T123F, T123Y and T123H, resulted in a further 12–14% higher ee_P_ towards (1*S*, 2*S*)-2-azidocyclohexan-1-ol. In contrast, the obtained ee_P_ of variant T123G was similar to HheG wild-type. Thus, mutagenesis of amino acid T123 in HheG did not only yield variants with substantially increased apparent melting temperature and significantly increased activity, but even slightly higher stereoselectivity. This further emphasizes the exceptional role of position 123 in HheG.

The observed positive impact of mutations at position 123 on activity and enantioselectivity of HheG was not only evident in the conversion of cyclohexene oxide, but also in the ring opening of other epoxide substrates (see Supplementary Fig. S3).

### Structural analysis of variants T123W and T123G

**Figure 3:**
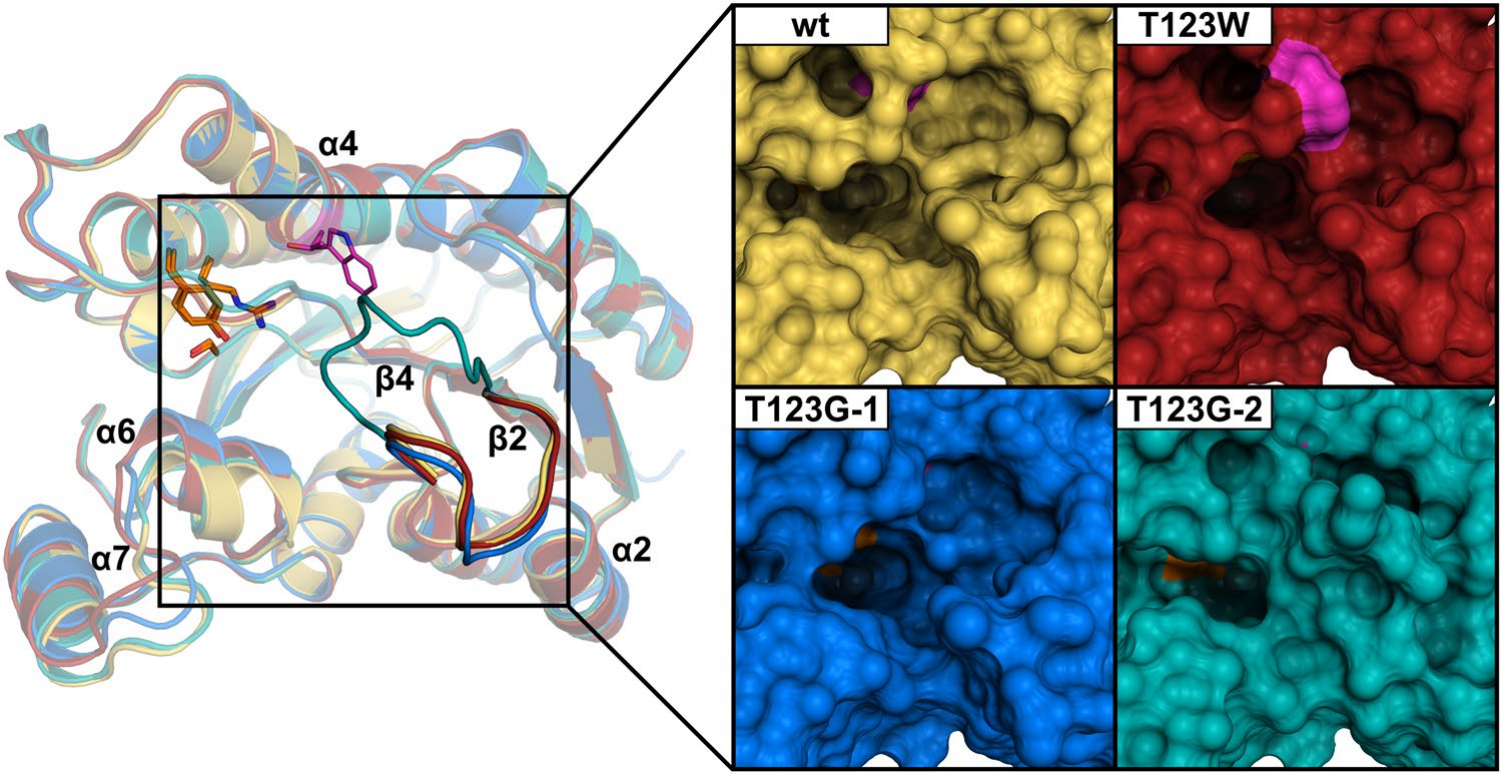
Left: Superimposed representative subunit structures of HheG wild-type (PDB entry: 5O30^17^, yellow) and its mutants (T123W: red; T123G_1: blue; T123G_2: teal) shown as cartoon representation. The catalytic triad (S152, Y165 and R169 in orange) and the mutated residue 123 (in magenta) are presented as sticks. Right: Surface representation of the active site cleft with the catalytic triad highlighted in orange and the side chain of residue 123 shown in magenta.

In order to derive a more detailed insight into the impact of stabilizing mutations T123G and T123W, we determined their crystal structures and compared them to the wild-type protein (PDB entry 5O30)^17^. Interestingly, whereas T123W yielded wild type-like crystals only, we obtained six different crystal forms for the T123G variant, which may already indicate an impact on the dynamic behavior of the protein. After solving and inspecting all of these crystal forms, two (T123G_1 and T123G_2) were chosen for final refinement and analysis, since they encompassed all structural changes with respect to the structure of the wild-type protein. Superimposition revealed that mutations T123G and T123W have no effect on the overall fold of the protein or on the position and orientation of the catalytic residues S152, Y165 and R169 (Supplementary Fig. S4). The active site cleft appears slightly more closed in some chains contained in the asymmetric units of the crystal forms described here, which is due to small movements of helices α6, α7 and the connecting loop (residues 200–222, Fig. 3). However, B-factor analysis revealed that this part exhibits considerable mobility in both mutants as well as in the wild-type structure, indicating that the observed shifts are not a direct consequence of mutation. More significantly, the flexible loop connecting strand β2 and helix α2 (residues 39 to 47) adopts a different conformation in three out of eight chains in the asymmetric unit of crystal form T123G_2. As a consequence, the loop moves towards residue 123 and leads to a partial covering of the active site cleft, resulting in significantly restricted access to the active site (Fig. 3). It must be noted that this different loop conformation is enforced by altered contacts in this crystal form as the position of the loop in its open conformation is occupied by crystallographic neighbors. However, to form a crystal in which the closed loop conformation is observable, this conformation has to be present during crystallization. Therefore, the T123G mutant favors the closed state more than the wild-type enzyme.

**Figure 4:**
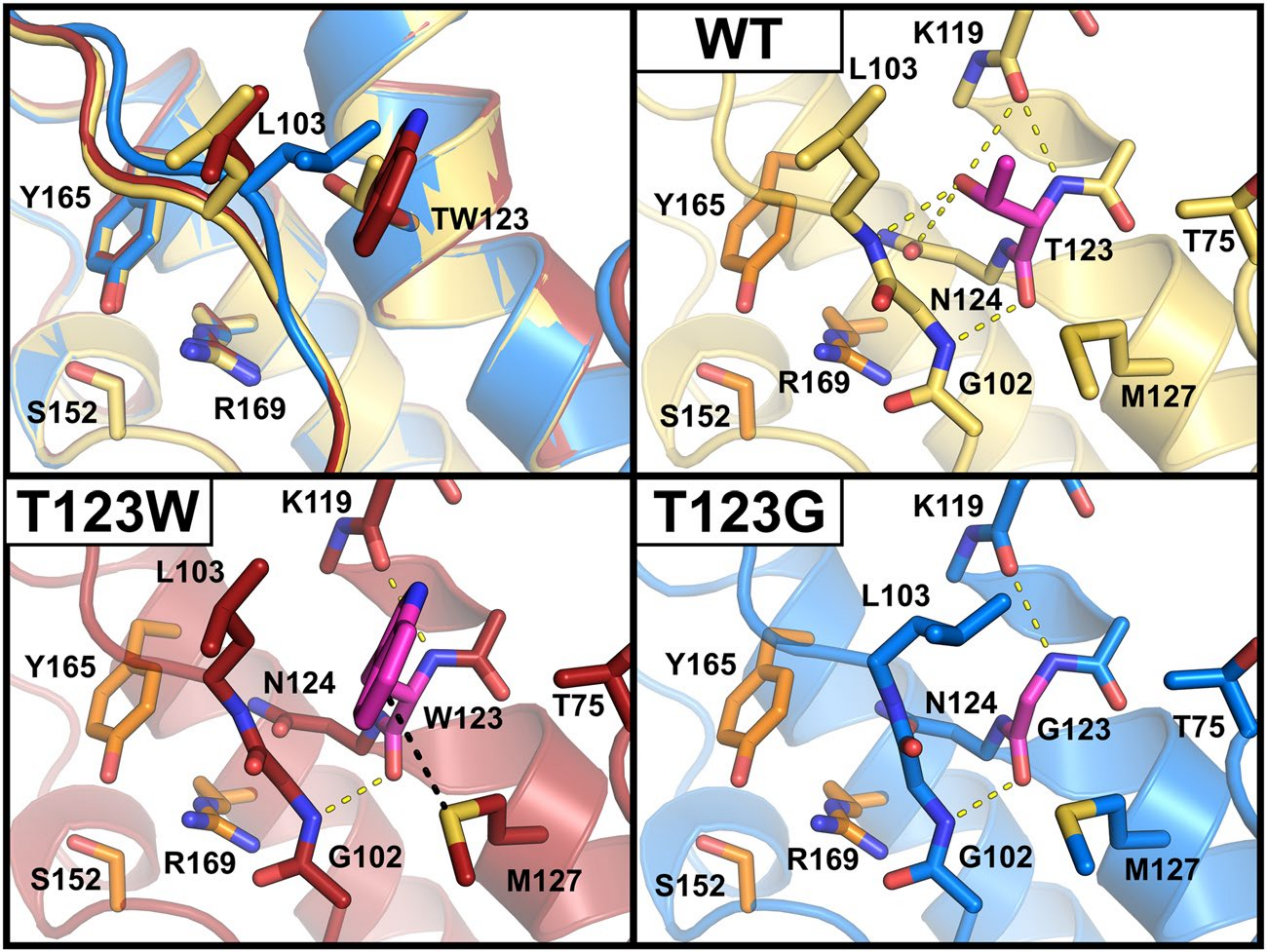
Close-up of the mutation site shown as cartoon and sticks representation with HheG wild-type shown in yellow, variant T123W in red and variant T123G in blue; catalytic residues highlighted in orange. Hydrogen bonds are presented as yellow dashed lines and sulfur-π interactions as black dashed lines. Only one of the structures of variant T123G is shown as they are nearly identical in regard to their interactions with residue 123 (highlighted in magenta).

A more thorough analysis of the variants shows that mutation of T123 to glycine or tryptophan leaves backbone hydrogen bonds to G102 and K119 unaltered, whereas side chain-specific contacts to L103, K119 and N124 are abolished (Fig. 4). In the T123W variant, new hydrophobic interactions of the tryptophan side chain with T75, L103 and M127 are established, which may contribute to the increased thermostability of this variant. Moreover, interactions between methionine and aromatic residues are known to have an even stronger stabilizing effect on proteins^25^, which might explain why the replacement of T123 with aromatic residues led to increased melting temperatures in the experiments reported above. In variant T123G, on the other hand, the additional space generated by the missing side chain is partially occupied by L103, resulting in a slight displacement of a loop connecting strand β4 and helix α4 (residues 101 to 107) that covers the catalytic triad. This intricate movement could impact on substrate positioning in the active site and may hence explain the increased catalytic activity of T123G.

### Molecular dynamics simulations

**Figure 5:**
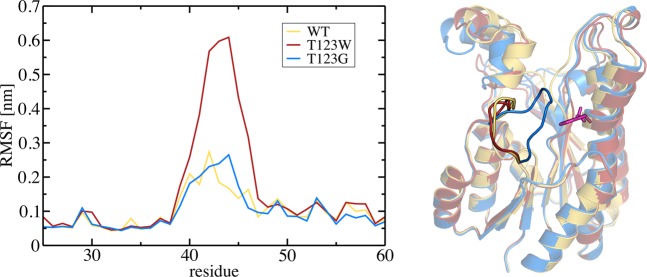
Left: RMSF plots for residues 25 to 60 from HheG wild-type (yellow), as well as variants T123W (red) and T123G (blue). Right: Superposition of average structures of one monomer taken from dimer MD simulations with the flexible loop highlighted in foreground. Colors as on the left; residue 123 is shown in magenta.

To further investigate the observed structural differences of the mutants, classical molecular dynamics (MD) simulations were performed. Dimers of HheG wild type as well as variants T123W and T123G were simulated for 250 ns, and root mean square fluctuations (RMSF) were calculated for the complete trajectories. RMSF is a measure for the deviation of atomic positions over a certain period of time and therefore correlates to B-factor values. This simulation analysis revealed significant differences in the mobility of the loop formed by residues 39 to 47 (Fig. 5, left), while the mobility of the remaining protein was unaffected. The loop of variant T123W displays a significantly higher mobility compared to wild-type HheG and variant T123G, for which a lower loop mobility has been obtained. As the RMSF analysis does not provide information on the absolute position of the loop, average structures over the simulation time were determined (Fig. 5, right). Here, a correlation between the average loop position and the amino acid at position 123 becomes obvious, whereas the remainder of the protein is virtually unchanged. The loop of HheG wild-type adopts a position similar to the one in the crystal structure, resulting in an open active site cleft. For T123W, the average loop position is shifted to slightly higher distances relative to residue 123. The most obvious change in loop position can be seen in variant T123G, where the loop is moved significantly toward residue 123, partially closing the active site cleft. Therefore, even though HheG wild-type and variant T123G display similar RMSF values, the average loop positions are very distinct. This partially closed conformation for T123G is in line with the observed loop conformation of some chains of the asymmetric unit of the T123G_2 crystal form described in the previous section (Fig. 3). In summary, the observed average loop position of variant T123W is very similar to the one found in HheG wild-type, while the loop mobility is significantly increased in this variant. Hence, also the ability of the T123W loop to (partially) close the active site cleft is increased compared to wild-type HheG. On the other hand, the T123G variant shows the closed active site conformation already in the average structure obtained from MD simulation. At the same time, its loop mobility is significantly lower compared to variant T123W. Therefore, both variants exhibit a significantly increased probability of adopting a (partially) closed active site conformation compared to HheG wild-type.

**Figure 6:**
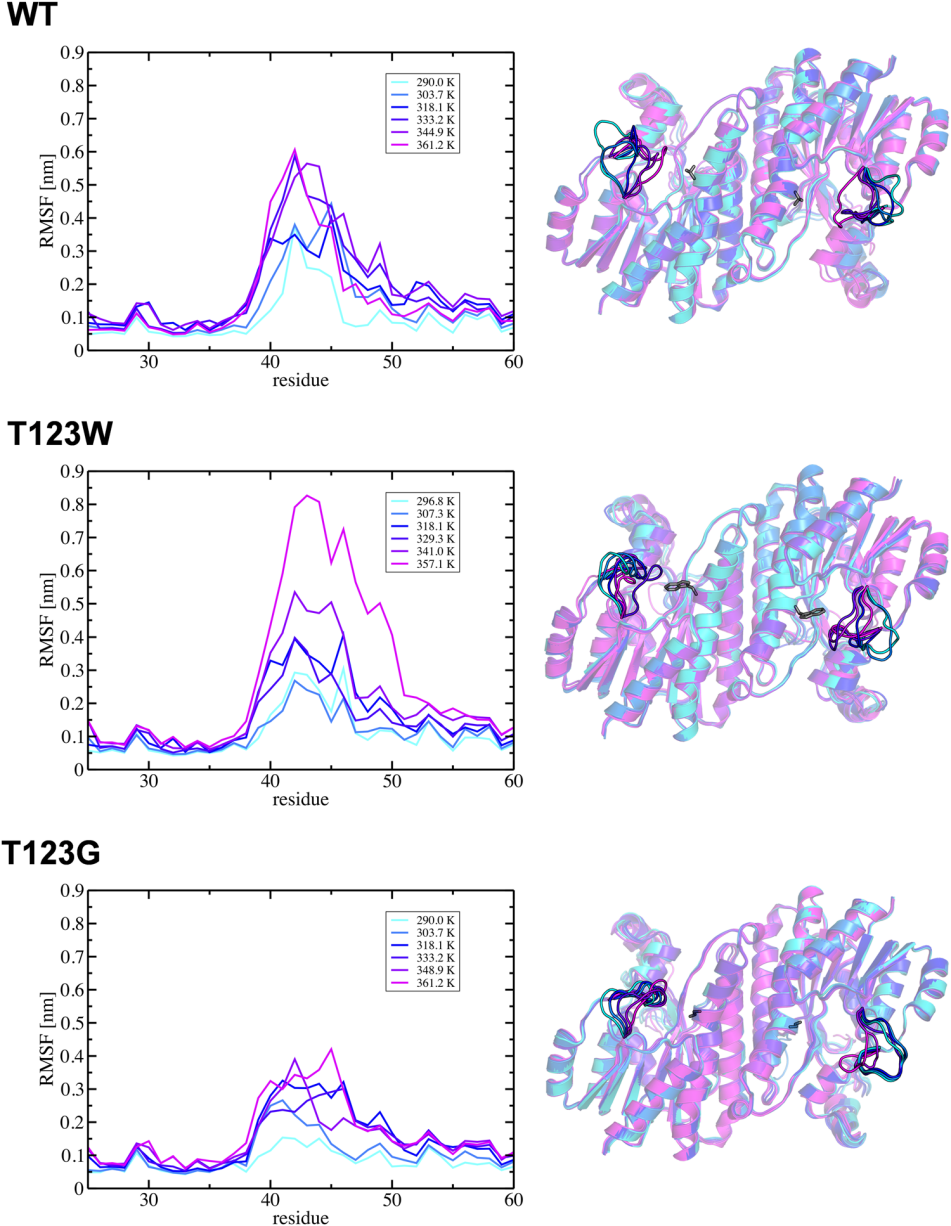
Left: RMSF plots for different temperatures (temperature increase from cyan over blue to magenta) taken from HREX simulations of HheG wild-type and variants T123W and T123G (data shown for one monomer). For all variants, the RMSF of the loop increases with increasing temperature. The smallest increase is observed for variant T123G. Right: Corresponding average dimer structures of wild-type HheG as well as variants T123W and T123G. Residue 123 is shown in grey.

As position and mobility of the loop present the main difference between the variants and wild-type HheG, a direct connection to the observed thermostability was assumed. To gain further insight into this, Hamiltonian replica exchange (HREX) simulations were performed, in which a system is simulated at several temperatures in parallel^26^. As expected, the mobility of the loop generally increases with increasing temperature (Fig. 6). However, this increase in mobility differs between the two variants and wild-type HheG. It is especially prominent for wild-type HheG and variant T123W. Here, the loop moves toward residue 123 with increasing temperature, resulting in larger RMSF values due to a partial closure of the active site cleft. In contrast, the loop mobility of variant T123G is less affected at higher temperatures, as the partially closed conformation of the loop is already observed in simulations at room temperature. Note that the HREX simulations were performed on dimers taken from the respective crystal structures, which display an open active site cleft. Therefore, the low temperature structures of T123G in Fig. 6 do not show the same loop position as the average structure shown in Fig. 5. Moreover, the HREX simulations were performed on significantly shorter time scale than the classical MD simulations at room temperature. Therefore, the large mobility of the loop in variant T123W observed in the room temperature simulations (Fig. 5, left) could not be accessed at the same temperature in our HREX simulations (Fig. 6, left), but was evident at higher temperatures.

Further analysis of HREX results revealed an unusual volume decrease (density increase) of the proteins with increasing temperature (Supplementary Fig. S5). This can be explained by the temperature-dependent loop movement toward residue 123, resulting in a denser packing of the protein. A dense packing was previously reported to contribute to the thermal stability of proteins^27^. Additionally, this loop movement is accompanied by an increase of buried surface area as parts of the active site cleft get shielded from the solvent. An increase in buried surface area is correlated to lower protein flexibility^28^, and hence higher stability. Improved thermostability of a halohydrin dehalogenase variant through an increase in buried surface area was already reported for HheC-2360^29^.

Together, the MD simulations indicate that two different mechanisms are responsible for the increased thermal stability of variants T123W and T123G. While the loop consisting of residues 39 to 47 leads to denser packing and increased buried surface area in T123G, it is more flexible in T123W. For the latter variant, the crystal structure indicates specific new interactions of the tryptophan with nearby residues, which might contribute to a higher thermostability of T123W. Moreover, the flexible loop could provide M45 as additional interaction partner for W123.

## Conclusions

Protein engineering of halohydrin dehalogenase HheG for thermostabilization revealed several residues with positive impact on the apparent melting temperature. For several variants, this gain in thermostability was accompanied by a loss in activity. As an exception, all variants at position 123 that exhibited a significantly higher T_m_, displayed also an increase in activity. Variant T123G was the most active one with a 5-fold higher initial reaction velocity in the azidolysis of cyclohexene oxide compared to wild-type HheG. In contrast, exchange of T123 by an aromatic residue yielded the highest gain in thermostability with 12–14 °C increase in T_m_. Crystal structures and MD simulations of variants T123W and T123G in comparison to wild-type HheG revealed a highly mobile loop close to the active site cleft, which can either adopt an open position or partially cover the cleft. In the latter case, the enzyme active site is more shielded from solvent and less accessible, as found in crystal structures of other known HHDHs^12–14^. Moreover, our data indicate that loop mobility and position are directly influenced by the amino acid at position 123.

Such large loop movements around the active site, as observed for HheG wild-type and its variants, have not been reported for other HHDHs so far. This further exemplifies the special position of HheG within the HHDH enzyme family. Additionally, this loop movement might be relevant for substrate binding in HheG.

MD simulations revealed that the thermostabilizing effect of mutations T123W and T123G is likely based on two distinct molecular mechanisms. Further research will be necessary to investigate this in more detail and to study the possible impact of tetramerization on the observed enzyme stability.

## Methods

### HheG engineering

Based on HheG’s crystal structure (PDB: 5O30^17^), several amino acid residues were selected for mutagenesis based on different in silico approaches: (i) residues displaying high B-factor values, (ii) possible introduction of additional hydrogen bonds in flexible loops and (iii) additional intersubunit interactions, (iv) the addition of hydrophilic amino acids on the enzyme’s surface and (v) residues identified by the Fireprot web server^23^. Depending on the approach, either focused mutant libraries were generated or point mutations were introduced (Table S1).

Site saturation mutagenesis (SSM) libraries and point mutations were generated by Quikchange® PCR using mutagenic primers and the 2×*PfuUltra* II Hotstart mastermix (Agilent Technologies, Santa Clara, CA, United States) according to the manufacturer’s instructions with minor modifications (see Supplementary for detailed information).

### Protein expression and purification in 96-well format

For library expression in 96-well plates, 300 µL TB medium supplemented with 50 mg L^−1^ kanamycin per well were inoculated from a master plate (see Supplementary). Resulting plates were incubated overnight at 37 °C and 700 rpm. From each well, 100 µL of this pre-culture were added to 1 mL fresh TB medium supplemented with 50 mg L^−1^ kanamycin and 0.2 mM isopropyl-β-D-thiogalactopyranoside (IPTG) as inducer in 96-deep-well plates. Expression was carried out at 22 °C and 1000 rpm for 24 h. Cells were harvested by centrifugation (3488 g, 20 min, 4 °C) and resulting cell pellets were stored at −20 °C until further use.

Purification of HheG mutants from the libraries was performed in 96-well format using His MultiTrap FF plates (GE Healthcare Life Sciences, Freiburg Germany). For this, frozen cell pellets were re-suspended in each 200 µL lysis buffer (50 mM Tris/SO_4_, 300 mM Na_2_SO_4_, 25 mM imidazole, pH 7.9, 1 mg mL^−1^ lysozyme, 100 µM phenylmethylsulfonyl fluoride (PMSF)) and incubated for 1 h at 30 °C and 700 rpm. The plate was frozen again at −20 °C for 30 min and afterwards thawed at 30 °C and 700 rpm. Then, 50 µL DNase solution (0.1 mg mL^−1^ DNase in 20 mM MgSO_4_) was added and the plate was incubated for another 30 min at 30 °C and 700 rpm. To obtain cell-free extract (CFE), the plate was centrifuged for 60 min at 3488 g and 4 °C. Purification of mutant enzymes from this CFE using His MultiTrap FF plates was carried out according to the manufacturer’s instructions. Elution of purified proteins was performed with 200 µL elution buffer (50 mM Tris/SO_4_, 300 mM Na_2_SO_4_, 400 mM imidazole, pH 7.9) per well, and 30 µL protein solution from each well was used for thermostability measurements (thermofluor assay) without prior desalting.

### Thermofluor assay

Apparent melting temperatures (T_m_) of enzymes were determined by thermal unfolding using the thermofluor assay^24^. The 50 µL reaction mixture contained 5x SYPRO Orange (Thermo Fisher Scientific, Waltham, MA, USA) and 30 µL purified but not desalted protein in elution buffer (in case of library screening) or 0.2 mg mL^−1^ purified and desalted protein in TE buffer (point mutants) in a iQ 96-well real-time PCR plate (Bio-Rad Laboratories, Munich, Germany). The plate was sealed and measured in a RT-PCR machine (Bio-Rad Laboratories, Munich, Germany, CFX96™ Real-Time PCR Detection Systems) over a linear gradient from 10 to 90 °C in 0.5 °C steps. The final T_m_ was derived from the local minimum of the negative first derivative of the measured relative fluorescence plotted versus the temperature.

### Protein crystallography

Protein crystals of N-terminal hexahistidine-tagged HheG variants T123G and T123W were obtained with the sitting drop vapor diffusion method at room temperature in 96-well INTELLI-Plates (Art Robbins Instruments, Sunnyvale, CA, USA). 200 nL of a solution containing 4 to 9 mg mL^−1^ of the respective HheG variant in 10 mM Tris/SO_4_ pH 8, 2 mM EDTA and 5 mM β-mercaptoethanol were mixed with 200 nL of reservoir solution utilizing a pipetting robot (Honeybee 963, Genomic Solutions, Huntingdon, U.K) and then equilibrated against 60 µL of reservoir solution. Commercially available sparse matrix screening suites were used for the identification of initial crystallization conditions, which were then improved by grid and random screening with precipitants prepared with a pipetting robot (Formulator, Formulatrix, Bedford, MA, United States). Protein crystals were fished from the crystallization drop with nylon loops, cryoprotected by addition of 10% (v/v) 2,3-(*R*,*R*)-butanediol to the reservoir solution and flash-cooled in liquid nitrogen. Collection of diffraction images was done at 100 K at beamline P11 at PETRAIII of the Deutsches Elektronen-Synchrotron (DESY, Hamburg, Germany)^30^ or at the beamline X06DA (PXIII) at the Swiss Light Source of the Paul Scherrer Institute (PSI, Villigen, Switzerland) utilizing a PILATUS 6M-F or a PILATUS 2M-F hybrid-pixel detector (DECTRIS Ltd., Baden-Daettwil, Switzerland), respectively. Crystallization conditions, protein concentration and the beamline used for collection of each data set are listed in Supplementary Table S4.

### Structure determination

Reflection image processing was performed using DIALS^31^, POINTLESS^32^ and AIMLESS^33^ of the CCP4 suite^34^. Initial phases were obtained by molecular replacement using PHASER^35^ and the atomic coordinates of HheG wild-type (PDB: 5O30^17^). Refinement was performed by alternating rounds of REFMAC5^36^ and manual adjustments in COOT^37^. Final refinement steps were done with phenix.refine^38^ of the PHENIX software suite^39^ implementing Translation/Liberation/Screw refinement^40^. MolProbity^41^ was used for structure validation. Diffraction data and coordinates were deposited in the Protein Data Bank^42^ (PDB entries: 6I9U; 6I9V; 6I9W). Representations of the structures were generated with PyMOL Molecular Graphics System version 2.1.1 (Schrödinger, LLC, New York, NY, USA). Data collection and refinement statistics are listed in Supplementary Table S4.

### Molecular dynamics simulations

MD simulations were performed using GROMACS 5.1.4^43^ and the Amber99SB-ILDN forcefield^44^ according to a standard protocol. Detailed information is provided in Table S5 in the supplementary. Hamiltonian replica exchange simulations were performed by GROMACS with PLUMED 2.3.5^45^ implementation using 20 replicas (Table S5). Analysis of RMSF values per residue as well as of protein volume and density was performed using GROMACS tools. PyMOL Molecular Graphics System version 2.1.1 was used for visualization and superposition of structures.

## Supplementary information

Supporting information

## Data Availability

The datasets generated during the current study, which are not included in this manuscript and its supplementary file or accessible via the PDB, are available from the corresponding author on reasonable request.

## References

[1] Schallmey A, Schallmey M (2016). Recent advances on halohydrin dehalogenases: from enzyme identification to novel biocatalytic applications. Appl. Microbiol. Biotechnol..

[2] Hasnaoui-Dijoux G, Majeric Elenkov M, Lutje Spelberg JH, Hauer B, Janssen DB (2008). Catalytic promiscuity of halohydrin dehalogenase and its application in enantioselective epoxide ring opening. ChemBioChem.

[3] Fox RJ (2007). Improving catalytic function by ProSAR-driven enzyme evolution. Nat. Biotechnol..

[4] Ma SK (2010). A green-by-design biocatalytic process for atorvastatin intermediate. Green Chem..

[5] Wan N-W (2015). An efficient high-throughput screening assay for rapid directed evolution of halohydrin dehalogenase for preparation of β-substituted alcohols. Appl. Microbiol. Biotechnol..

[6] Yao P (2015). Efficient biosynthesis of ethyl (*R*)-3-hydroxyglutarate through a one-pot bienzymatic cascade of halohydrin dehalogenase and nitrilase. ChemCatChem.

[7] van Hylckama Vlieg JET (2001). Halohydrin dehalogenases are structurally and mechanistically related to short-chain dehydrogenases / reductases. J. Bacteriol..

[8] Kavanagh KL, Jörnvall H, Persson B, Oppermann U (2008). The SDR superfamily: Functional and structural diversity within a family of metabolic and regulatory enzymes. Cell. Mol. Life Sci..

[9] Yu F, Nakamura T, Mizunashi W, Watanabe I (1994). Cloning of two halohydrin hydrogen-halide-lyase genes of *Corynebacterium* sp. strain N-1074 and structural comparison of the genes and gene products. Biosci. Biotechnol. Biochem..

[10] Poelarends GJ, van Hylckama Vlieg JET, Marchesi JR, Freitas Dos Santos LM, Janssen DB (1999). Degradation of 1,2-dibromoethane by *Mycobacterium* sp. strain GP1. J. Bacteriol..

[11] Higgins TP, Hope SJ, Effendi AJ, Dawson S, Dancer BN (2005). Biochemical and molecular characterisation of the 2,3-dichloro-1-propanol dehalogenase and stereospecific haloalkanoic dehalogenases from a versatile *Agrobacterium* sp. Biodegradation.

[12] Watanabe F (2015). Crystal structures of halohydrin hydrogen-halide-lyases from *Corynebacterium* sp. N-1074. Proteins Struct. Funct. Bioinforma..

[13] de Jong RM, Kalk KH, Tang L, Janssen DB, Dijkstra BW (2006). The X-ray structure of the haloalcohol dehalogenase HheA from *Arthrobacter* sp. strain AD2: Insight into enantioselectivity and halide binding in the haloalcohol dehalogenase family. J. Bacteriol..

[14] de Jong RM (2003). Structure and mechanism of a bacterial haloalcohol dehalogenase: a new variation of the short-chain dehydrogenase/reductase fold without an NAD(P)H binding site. EMBO J..

[15] Schallmey M, Koopmeiners J, Wells E, Wardenga R, Schallmey A (2014). Expanding the halohydrin dehalogenase enzyme family: Identification of novel enzymes by database mining. Appl. Environ. Microbiol..

[16] Koopmeiners J, Halmschlag B, Schallmey M, Schallmey A (2016). Biochemical and biocatalytic characterization of 17 novel halohydrin dehalogenases. Appl. Microbiol. Biotechnol..

[17] Koopmeiners J (2017). HheG, a halohydrin dehalogenase with activity on cyclic epoxides. ACS Catal..

[18] Vaidyanathan R, Hesmondhalgh L, Hu S (2007). A chemoenzymatic synthesis of an androgen receptor antagonist. Org. Process Res. Dev..

[19] Sharma A, Agarwal J, Peddinti RK (2017). Direct access to the optically active VAChT inhibitor vesamicol and its analogues *via* the asymmetric aminolysis of *meso*-epoxides with secondary aliphatic amines. Org. Biomol. Chem..

[20] Bommarius AS, Paye MF (2013). Stabilizing biocatalysts. Chem. Soc. Rev..

[21] Arabnejad H (2017). A robust cosolvent-compatible halohydrin dehalogenase by computational library design. Protein Eng. Des. Sel..

[22] Wu Z (2017). Exploring the thermostable properties of halohydrin dehalogenase from *Agrobacterium radiobacter* AD1 by a combinatorial directed evolution strategy. Appl. Microbiol. Biotechnol..

[23] Bednar D (2015). FireProt: Energy- and evolution-based computational design of thermostable multiple-point mutants. PLoS Comput. Biol..

[24] Ericsson UB, Hallberg BM, DeTitta GT, Dekker N, Nordlund P (2006). Thermofluor-based high-throughput stability optimization of proteins for structural studies. Anal. Biochem..

[25] Valley CC (2012). The methionine-aromatic motif plays a unique role in stabilizing protein structure. J. Biol. Chem..

[26] Bussi G (2014). Hamiltonian replica exchange in GROMACS: A flexible implementation. Mol. Phys..

[27] Vogt G, Argos P (1997). Protein thermal stability: Hydrogen bonds or internal packing?. Fold. Des..

[28] Marsh JA (2013). Buried and accessible surface area control intrinsic protein flexibility. J. Mol. Biol..

[29] Schallmey M (2013). Biocatalytic and structural properties of a highly engineered halohydrin dehalogenase. ChemBioChem.

[30] Burkhardt A (2016). Status of the crystallography beamlines at PETRA III. Eur. Phys. J. Plus.

[31] Winter G (2018). DIALS: Implementation and evaluation of a new integration package. Acta Crystallogr. Sect. D Struct. Biol..

[32] Evans PR (2011). An introduction to data reduction: space-group determination, scaling and intensity statistics. Acta Crystallogr. Sect. D Biol. Crystallogr..

[33] Evans PR, Murshudov GN (2013). How good are my data and what is the resolution?. Acta Crystallogr. Sect. D Biol. Crystallogr..

[34] Collaborative Computational Project, N. 4 (1994). The CCP4 Suite: Programs for Protein Crystallography. Acta Crystallogr. Sect. D Biol. Crystallogr..

[35] McCoy AJ (2007). Phaser crystallographic software. J. Appl. Crystallogr..

[36] Murshudov GN (2011). REFMAC5 for the refinement of macromolecular crystal structures. Acta Crystallogr. Sect. D Biol. Crystallogr..

[37] Emsley P, Lohkamp B, Scott WG, Cowtan K (2010). Features and development of Coot. Acta Crystallogr. Sect. D Biol. Crystallogr..

[38] Afonine PV (2012). Towards automated crystallographic structure refinement with *phenix.refine*. Acta Crystallogr. Sect. D Biol. Crystallogr..

[39] Adams PD (2010). *PHENIX*: A comprehensive Python-based system for macromolecular structure solution. Acta Crystallogr. Sect. D Biol. Crystallogr..

[40] Schomaker V, Trueblood KN (1968). On the rigid-body motion of molecules in crystals. Acta Crystallogr. Sect. B Struct. Crystallogr. Cryst. Chem..

[41] Chen VB (2010). *MolProbity*: all-atom structure validation for macromolecular crystallography. Acta Crystallogr. Sect. D Biol. Crystallogr..

[42] Berman HM (2000). The Protein Data Bank. Nucleic Acids Res..

[43] Van Der Spoel D (2005). GROMACS: Fast, Flexible, and Free. J. Comput. Chem..

[44] Lindorff-Larsen K (2010). Improved side-chain torsion potentials for the Amber ff99SB protein force field. Proteins Struct. Funct. Bioinforma..

[45] Tribello GA, Bonomi M, Branduardi D, Camilloni C, Bussi G (2014). PLUMED 2: New feathers for an old bird. Comput. Phys. Commun..

