# Supplementary material for: Position 123 of halohydrin dehalogenase HheG plays an important role in stability, activity, and enantioselectivity
Source: Sci Rep. 2019 Mar 25;9:5106. doi: 10.1038/s41598-019-41498-2 (PMC6434027; doi:10.1038/s41598-019-41498-2)
Supplement: Supplementary file 1 — Supporting information [file 41598_2019_41498_MOESM1_ESM.pdf]

Supplementary Information

Position 123 of halohydrin dehalogenase HheG plays an important role in stability, activity, and enantioselectivity

Jennifer Solarczek[1], Thomas Klünemann[2], Felix Brandt[3], Patrick Schrepfer[1], Mario Wolter[3], Christoph R. Jacob[3], Wulf Blankenfeldt[2] and Anett Schallmey[1,*]

[1]Institute for Biochemistry, Biotechnology and Bioinformatics, Technische Universität Braunschweig, Spielmannstr. 7, 38106 Braunschweig, Germany

[2]Structure and Function of Proteins, Helmholtz Centre for Infection Research, Inhoffenstr. 7, 38124 Braunschweig, Germany

[3]Institute of Physical and Theoretical Chemistry, Technische Universität Braunschweig, Gaußstr. 17, 38106 Braunschweig, Germany

[*]Corresponding author:

Prof. Dr. Anett Schallmey
phone: +49 531 391-55400
fax: +49 531 391-55401
e-mail: a.schallmey@tu-braunschweig.de
ORCID: 0000-0002-6670-0574

HheG engineering

Table S1: Residues that have been selected by computational means for the generation of site-saturation mutagenesis libraries or single mutants of HheG to enhance its thermostability.

Strategy	Library	Single mutants
High B factor value	E214X<br>R208X<br>V120X	
Introduce buried hydrophobic interactions between subunits	A178X<br>D255X	G171A
Introduce new hydrogen bonds in flexible loops	D65X<br>R226X	
Fireprot webserver[11]	T123X<br>C147X	A144S<br>M161G<br>G174M<br>V46P
Hydrophilic surface		M45K/R<br>Q81K/R<br>S82K/R<br>E85K/R<br>G107K/R<br>D115K/R

Table S2: Determined apparent melting temperatures of double mutants and their deviation from the respective single mutant.

	Melting temperature [°C]	Deviation from single mutant C147I or T123W/H/Y [°C]
C147I_R208G	42	1
T123W_C147I	45	-7
T123W_R208G	49	-3
T123H_C147I	45	-7
T123H_R208G	48	-4
T123Y_C147I	47	-5
T123Y_R208G	49.5	-2.5

Characterization of HheG T123 single mutants

Table S3: Apparent melting temperatures of HheG wild-type (WT) and mutants T123F, T123G and T123W determined by CD spectroscopy.

	Melting temperature [°C]
HheG WT	38.0
T123F	49.0
T123G	44.5
T123W	51.3

To investigate if the increased apparent melting temperatures of T123 single mutants result also in higher temperature optima, which is not always the case,[1] temperature profiles of HheG variants T123W and T123G were determined in the azidolysis of cyclohexene oxide and compared to wild-type. HheG T123W was selected as this variant displayed a 14 °C higher T_m while activity was still most similar to HheG wild-type. Additionally, mutant T123G was chosen based on its slightly lower improvement in apparent melting temperature (ΔT_m = 7 °C) but significantly higher activity. In contrast to the sharp temperature optimum of HheG wild-type, mutants T123W and T123G display a broader temperature optimum, which is also shifted to higher temperatures (Figure S1). Thus, the mutants are also more thermostable under reaction conditions. Interestingly, mutant T123W still showed 65% relative activity at 4 °C, whereas the respective relative activity of wild-type HheG and mutant T123G dropped below 30% at this temperature.

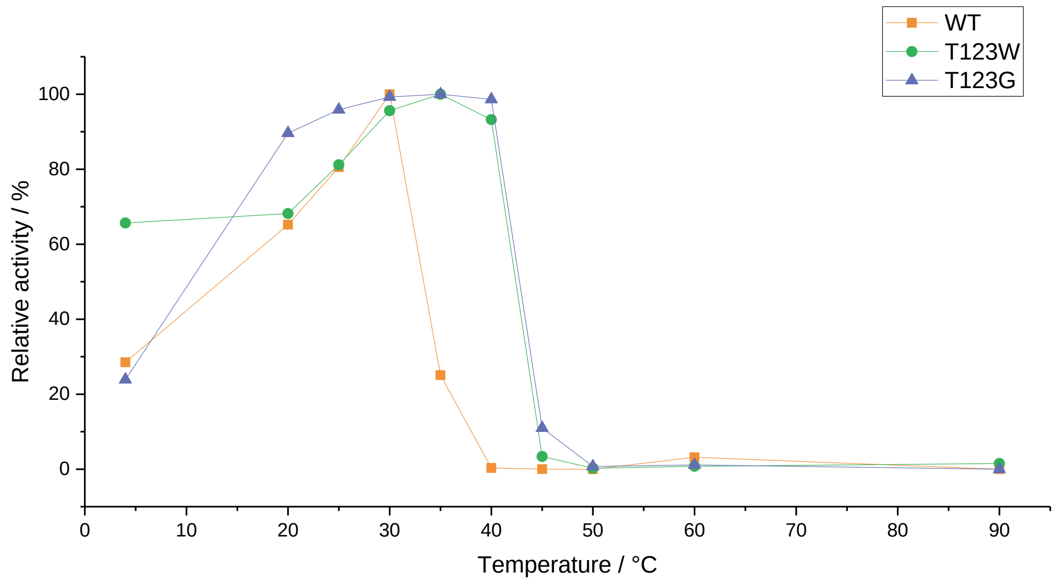

Figure S1: Temperature profiles of HheG wild-type (orange, square) and mutants T123W (green, circle) and T123G (purple, triangle).

In literature, it is often reported that thermostabilized mutants are also more resistant toward co-solvents compared to their wild-type counterparts.[2–6] Therefore, also the solvent resistance of variants T123W and T123G was studied in comparison to HheG wild-type. As mentioned earlier, the addition of a co-solvent in enzymatic reactions is often required to enhance substrate solubility during bioconversion. In many cases, DMSO is used for this purpose as it can dissolve a large variety of organic compounds, has low chemical reactivity and a low vapor pressure[7]. In our investigation of HheG wild-type and its mutants, however, DMSO was found to be the worst co-solvent among the tested ones, as a strong negative effect on enzyme activity was observed even at low concentrations (Figure S2). In contrast, the less polar co-solvent isopropanol was much better tolerated by HheG wild-type and mutant T123W yielding high residual activities even at 10% co-solvent concentration. Interestingly, the activity of variant T123G was similarly affected by all applied co-solvents as in all cases a similar activity decrease with increasing co-solvent concentration was observed. Only acetonitrile at low concentrations (1.8 and 5%) yielded slightly higher residual activities for this mutant compared to all other tested co-solvents. In case of mutant T123W, methanol and isopropanol even had a slight activating effect at the lowest tested co-solvent concentration. Such an activating effect (1.6-fold increase of activity) was also found for the thermostable variant H12 of HheC using 25 and 50% acetonitrile as co-solvent.[8] In our case, addition of 25% co-solvent concentration inactivated the tested enzymes almost completely, except for HheG wild-type and mutant

T123W with isopropanol as co-solvent. The latter is likely explained by the lower polarity of isopropanol compared to all other tested co-solvents.

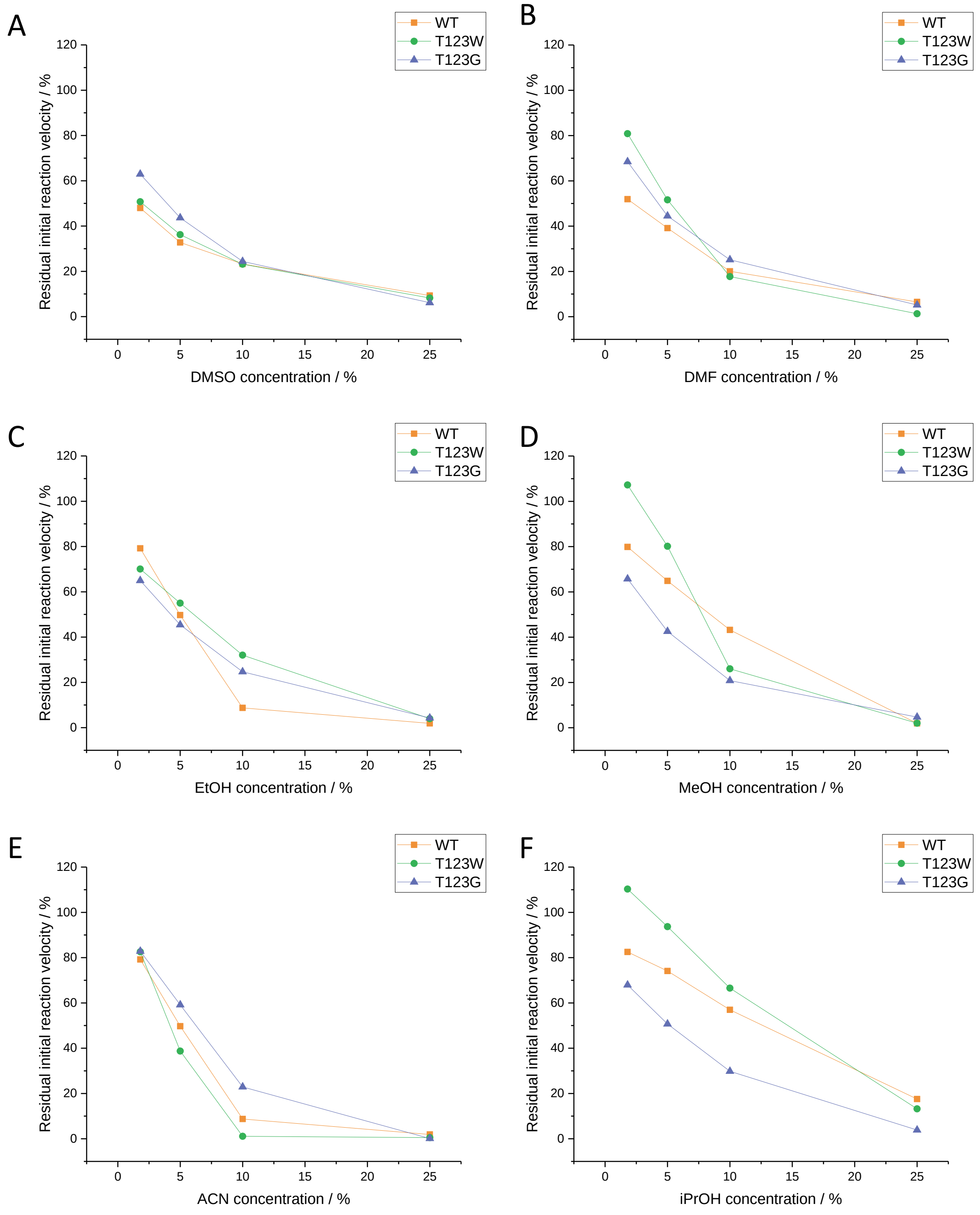

Figure S2: Residual initial reaction velocities of wild-type HheG (WT), mutant T123W and mutant T123G in the azidolysis of cyclohexene oxide with addition of different co-solvent concentrations: A) dimethyl sulfoxide (DMSO), B) dimethyl formamide (DMF), C) ethanol (EtOH), D) methanol (MeOH), E) acetonitrile (ACN), F) isopropanol (iPrOH). Initial reaction

velocities of HheG wild-type as well as variants T123W and T123G without addition of any co-solvent reached values of 0.222 ± 0.008 µmol min^{-1}, 0.363 ± 0.005 µmol min^{-1} and 0.83 ± 0.03 µmol min^{-1}, respectively.

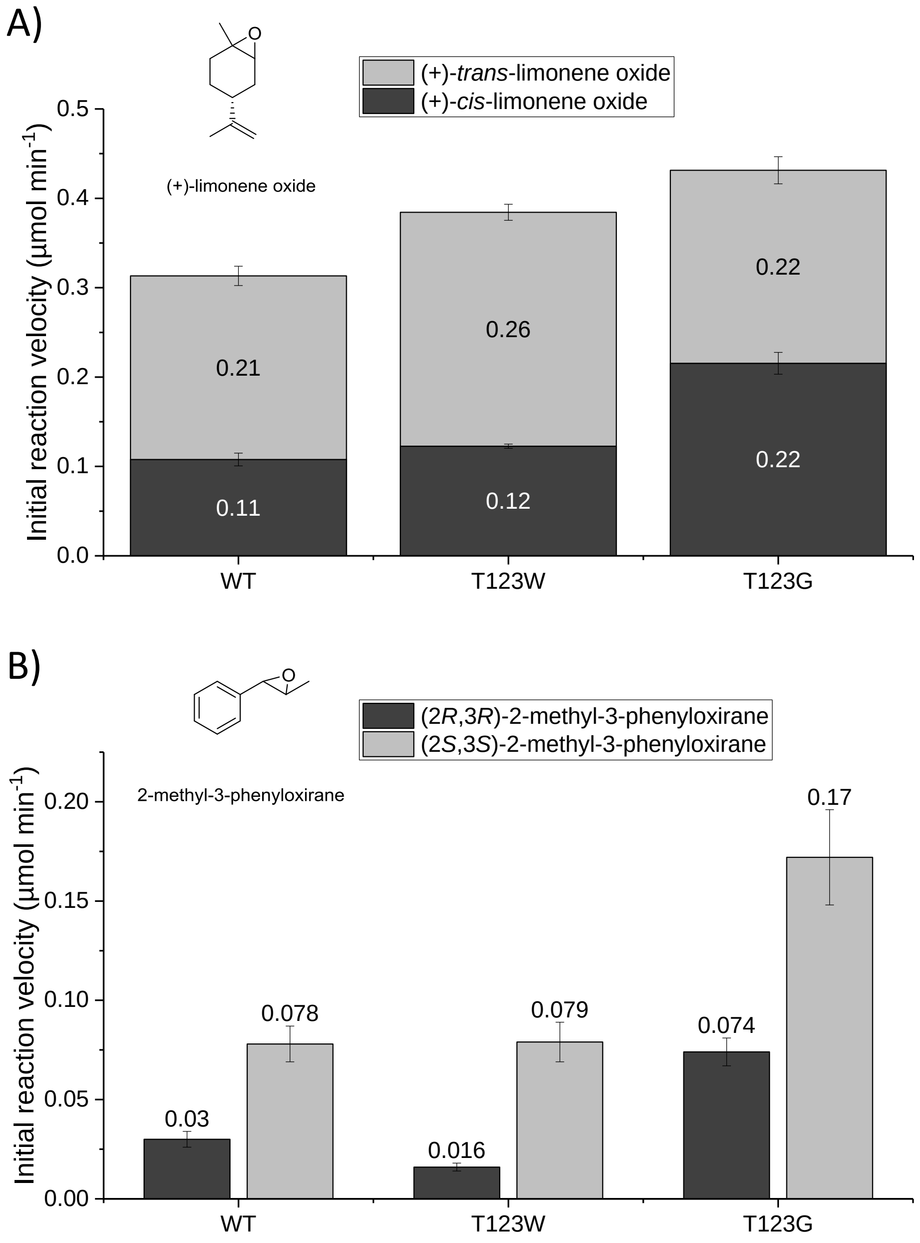

Figure S3: Initial reaction velocities of HheG wild-type as well as variants T123W and T123G with A) (+)-limonene oxide and B) (2*R*, 3*R*)- and (2*S*, 3*S*)-2-methyl-3-phenyloxirane.

Structural analysis of variants T123W and T123G

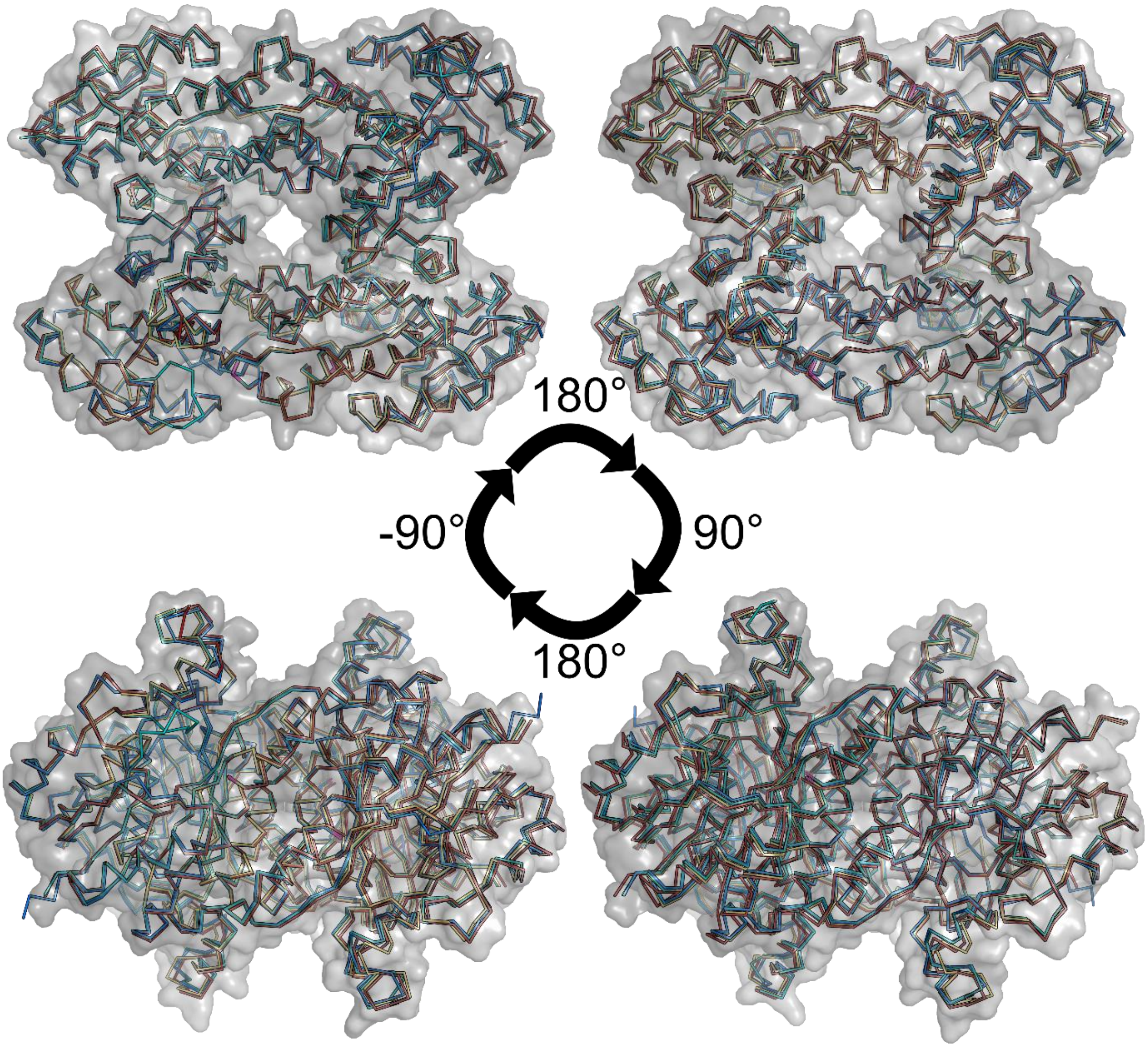

Figure S4: Top, bottom and side views of superimposed homotetramers of halohydrin dehydrogenase HheG from *Ilumatobacter coccineus* (PDB entry: 5O30[11], yellow) and its mutants (T123W: red; T123G_1: blue; T123G_2: teal) presented as C_α-traces. Additionally, wild-type HheG is represented as grey transparent surface. The RMSDs of the C_α atoms for the superimposed tetramers were calculated as 0.616Å (T123W), 0.639Å (T123G_1) and 0.615Å (T123G_2).

Table S4: Crystallization, data collection and refinement statistics for HheG variants

	**HheG_T123W (PDB: 6I9U)**	**HheG_T123G_1 (PDB: 6I9W)**	**HheG_T123G_2 (PDB: 6I9V)**
**Crystallisation**			
Condition	18.3% Glycerol	0.1M Tris/HCl pH8.5	0.2M Sodium thiocyanate
	11.1% PEG4000	25% PEG8000	20%PEG3350
	0.1M Imidazol pH 7.34		
Protein concentration (mg/ml)	9	4	4
Synchrotron	DESY	DESY	SLS
**Data Collection**			
Wavelength	1.000025	0.953714	1.000030
Resolution range[a]	49.34 - 2.4 (2.486 - 2.4)	53.71 - 1.55 (1.605 - 1.55)	109.3 - 2.8 (2.9 - 2.8)
Space group	P 31 2 1	P 1 21 1	P 21 21 2
Unit cell			
a; b; c (Å)	197.25; 197.25; 197.72	66.61; 107.42; 67.24	102.03; 141.32; 172.52
α; β; γ (°)	90; 90; 120	90; 90.97; 90	90; 90; 90
Total reflections[a]	1978343 (193880)	907682 (89085)	808428 (76103)
Unique reflections[a]	172776 (17132)	135646 (13439)	62175 (6135)
Multiplicity[a]	11.5 (11.3)	6.7 (6.6)	13.0 (12.4)
Completeness (%)[a]	99.93 (99.96)	99.17 (98.50)	99.71 (99.67)
I/σ(I)[a]	18.36 (1.18)	18.87 (1.85)	16.81 (1.44)
Wilson B-factor	56.75	15.68	50.87
R_{merge}[a]	0.1034 (2.311)	0.1183 (0.4905)	0.3759 (0.8275)
R_{meas}[a,b]	0.1083 (2.42)	0.1282 (0.5321)	0.3916 (0.864)
R_{pim}[a,c]	0.03184 (0.7148)	0.049 (0.2045)	0.1088 (0.2462)
$CC_{1/2}$[a,d]	0.999 (0.546)	0.994 (0.88)	0.947 (0.868)
CC*[a]	1 (0.841)	0.999 (0.967)	0.986 (0.964)
**Model Refinement**			
Reflections used in refinement[a]	172697 (17132)	135635 (13439)	61997 (6115)
Reflections used for R_{free}[a]	8503 (786)	6863 (672)	3080 (299)
R_{work}[a,e]	0.2013 (0.3263)	0.1402 (0.2341)	0.2213 (0.3343)
R_{free}[a,f]	0.2226 (0.3255)	0.1641 (0.2321)	0.2558 (0.3602)
CC_{work}[a]	0.481 (0.271)	0.970 (0.929)	0.899 (0.805)
CC_{free}[a]	0.460 (0.266)	0.961 (0.915)	0.921 (0.762)
Protein residues	2572	1032	15174
RMS(bonds)	0.002	0.008	0.002
RMS(angles)	0.48	0.84	0.46
Ramachandran favored (%)	96.12	98.05	94.59
Ramachandran allowed (%)	3.88	1.95	5.41
Ramachandran outliers (%)	0.00	0.00	0.00
Rotamer outliers (%)	0.05	0.00	0.00
Clashscore	2.95	2.10	4.14
Average B-factor	82.95	23.66	67.18
Number of TLS groups	60	14	43

[a]Values in parentheses are for the highest resolution shell.

${}^{b}R_{meas} = \Sigma_{hkl}\{N(hkl)/[N(hkl) - 1]\}^{1/2} \times \Sigma_i|I_i(hkl) - \langle I(hkl)\rangle|/\Sigma_{hkl}\Sigma_i I_i(hkl)$.
${}^{c}R_{pim} = \Sigma_{hkl}\{1/[N(hkl) - 1]\}^{1/2} \times \Sigma_i|I_i(hkl) - \langle I(hkl)\rangle|/\Sigma_{hkl}\Sigma_i I_i(hkl)$.
${}^{d}CC_{1/2} = \Sigma(x - \langle x\rangle)(y - \langle x\rangle)/[\Sigma(x - \langle x\rangle)^2\Sigma(y - \langle y\rangle)^2]^{1/2}$.
${}^{e}R_{work} = (\Sigma_{hkl}||F_{obs}| - k|F_{calc}||)/(\Sigma_{hkl}|F_{obs}|)$.
${}^{f}R_{free}$ is the same as R_{work} with 5% of reflections chosen at random and omitted from refinement.
Statistics were calculated with the Table 1 tool of PHENIX suite.[12] All Data sets were collected from single crystals at PetraIII at the German electron synchrotron (DESY, Hamburg, Germany) and the Swiss Light Source (SLS) at the Paul Scherrer Institute (PSI, Villigen, Switzerland).

Molecular dynamics simulations

Table S5: Simulation parameters for MD and HREX simulations of HheG dimers.

Simulation	Simulation parameters	
**MD simulations**	Preparation	solvation in water box, neutralization, minimization, NVT (100 ps), NPT (100 ps)
	Steps	125,000,000
	Time per step	2 fs
	Temperature	293 K
	Temperature coupling	Nosé-Hoover
	Pressure coupling	Parrinello-Rahman
	Constraints	LINCS
	Coordinate output every	5,000 steps
**Hamiltonian replica exchange simulations**	Preparation	start from structure after NPT run (see MD)
	Steps	15,000,000
	Time per Step	2 fs
	Exchange attempt every	200 steps
	Temperature range (simulation)	290 to 450 K
	Temperature range (real)	290 to 361 K
	Temperature coupling	V-rescale
	Pressure coupling	no
	Constraints	LINCS
	Coordinate output every	5000 steps

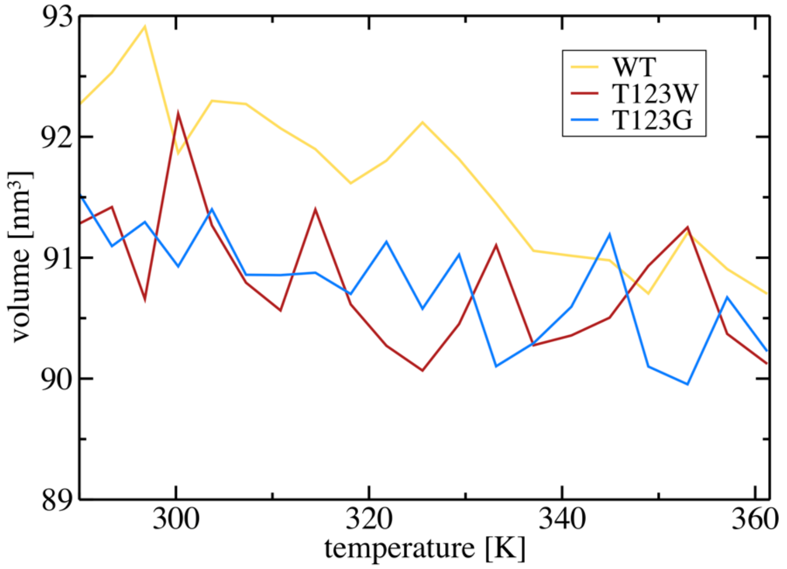

Figure S5: Observed volume decrease with increasing temperature in the simulation of dimers of HheG wild type and mutants T123W and T123G.

Methods

Chemicals

All chemicals were of analytical grade or the highest available purity. Substrate cyclohexene oxide was purchased from Acros Organics. (+)-Limonene oxide, (2*R*, 3*R*)- and (2*S*, 3*S*)-2-methyl-3-phenyloxirane were obtained from Sigma Aldrich.

Bacterial strains and plasmids

E. coli XL-1 blue (Thermo Fisher Scientific, Waltham, MA, USA) was used for library cloning. *E. coli* BL21 (DE3) Gold (Life Technologies, Darmstadt, Germany) was used as host for library screening and heterologous protein production. pET28a(+)-based vectors harboring a T7-promoter and resulting in N-terminal hexahistidine (His_6) tag fusion were used for expression of HheG wild-type and mutant genes.

HheG engineering

Mutagenic PCR reactions contained 25 µL 2x *PfuUltra* II Hotstart Mastermix, 100 ng pET28a(+)hheG and each 0.1 µM forward and reverse primer in a total volume of 50 µL. For the generation of SSM libraries, mutagenic primers containing the degenerated NNS codon (Table S6) were used in the PCR mixture. After PCR amplification, parental DNA was digested

at 37 °C overnight using 10 U DpnI. The PCR mix was used to transform chemically competent *E. coli* XL-1 blue (in case of SSM) or BL21 (DE3) Gold (for point mutations) cells and plated on LB agar containing 50 mg L^{-1} kanamycin. In case of single amino acid exchanges, plasmid DNA of single colonies was isolated using the E.Z.N.A. Plasmid DNA Mini Kit I (Omega Bio-Tek Inc, Norcross, GA, USA). For SSM libraries, all obtained *E. coli* XL-1 blue colonies were combined and plasmid DNA was isolated. Successful mutagenesis was confirmed by sequencing (Eurofins Genomics, Ebersberg, Germany). 50 – 100 ng plasmid DNA mix (SSM libraries) was then used to transform 50 µL chemically competent *E. coli* BL21 (DE3) Gold cells. Afterwards, 94 single clones per library were picked and transferred into a 96-well microtiter plate (MTP) containing 300 µL TB medium supplemented with 50 mg L^{-1} kanamycin per well. As controls, *E. coli* BL21 (DE3) Gold cells containing pET28a(+)hheG (wild-type) and pET28a(+) (empty vector control) were added in wells A1 and H12, respectively. Plates were incubated overnight at 37 °C and 700 rpm in an MTP shaker (VWR). From each well, 100 µL of the overnight culture were added to 100 µL 50% (v/v) sterile glycerol in a fresh MTP and the resulting master plates were stored at -80 °C until further use.

Table S6: List of applied mutagenic oligonucleotides for HheG engineering. Mutagenesis sites are shown in bold.

**Name**	**Nucleotide sequence (5' → 3')**
E85R_fwd<br>E85R_rev	GGTAATCAGAGCATGATT**CGT**CGTGTTCTGGAACGTTTTG<br>CAAAACGTTCCAGAACACG**ACG**AATCATGCTCTGATTACC
E85K_fwd<br>E85K_rev	GTAATCAGAGCATGATT**A**AACGTGTTCTGGAACG<br>CGTTCCAGAACACGTT**T**AATCATGCTCTGATTAC
S82R_fwd<br>S82R_rev	CCCGTACCGGTAATCAG**CGT**ATGATTGAACGTGTTC<br>GAACACGTTCAATCAT**ACG**CTGATTACCGGTACGGG
S82K_fwd<br>S82K_rev	CCGTACCGGTAATCAGA**AA**ATGATTGAACGTGTTCTG<br>CAGAACACGTTCAATCAT**TT**TCTGATTACCGGTACGG
D115R_fwd<br>D115R_rev	CTGGATATGACCGAT**CG**TCAGTGGGCAAAAG<br>CTTTTGCCCACTGA**CG**ATCGGTCATATCCAG
D115K_fwd<br>D115K_rev	TCTGGATATGACCGAT**AAA**CAGTGGGCAAAAGTTAA<br>TTAACTTTTGCCCACTG**TTT**ATCGGTCATATCCAGA
Q81R_fwd<br>Q81R_rev	CCCGTACCGGTAATC**GT**AGCATGATTGAACG<br>CGTTCAATCATGCT**AC**GATTACCGGTACGGG
Q81K_fwd<br>Q81K_rev	GACCACCCGTACCGGTAAT**AAA**AGCATGATTGAACGTGTTC<br>GAACACGTTCAATCATGCT**TTT**ATTACCGGTACGGGTGGTC
G107R_fwd<br>G107R_rev	GACCGGTCTGATTGTTACC**CGT**AAATTTCTGGATATGACC<br>GGTCATATCCAGAAATTT**ACG**GGTAACAATCAGACCGGTC

G107K_fwd<br>G107K_rev	GACCGGTCTGATTGTTACC**AAA**AAATTTCTGGATATGACC<br>GGTCATATCCAGAAATTT**TTT**GGTAACAATCAGACCGGTC
M45R_fwd<br>M45R_rev	CAGCCGGTGATGGCACC**CGT**GTTGGTGTTGAAGAAAG<br>CTTTCTTCAACACCAAC**ACG**GGTGCCATCACCGGCTG
M45K_fwd<br>M45K_rev	GCCGGTGATGGCACCA**AA**GTTGGTGTTGAAGAAAG<br>CTTTCTTCAACACCAAC**TT**TGGTGCCATCACCGGC
G171A_fwd<br>G171A_rev	TGGCACCCGTGCCG**CG**GCAAATGGTATTGT<br>ACAATACCATTTGC**CG**CGGCACGGGTGCCA
V120_NNS_fwd<br>V120_NNS_rev	CCGATGATCAGTGGGCAAAA**NNS**AAAGCAACCAACCTGGATATG<br>CATATCCAGGTTGGTTGCTTT**SNN**TTTTGCCCACTGATCATCGG
E214_NNS_fwd<br>E214_NNS_rev	GCAGATGGTGATCCG**NNS**CGTCGTGCAATGATTG<br>CAATCATTGCACGACG**SNN**CGGATCACCATCTGC
R208_NNS_fwd<br>R208_NNS_rev	GGTTTTCTGAAAGCAAGC**NNS**GCAGATGGTGATCCGGAAC<br>GTTCCGGATCACCATCTGC**SNN**GCTTGCTTTCAGAAAACC
A178_NNS_fwd<br>A178_NNS_rev	GTGCAAATGGTATTGTTCGT**NNS**GTTGGTCTGGAACATGCACG<br>CGTGCATGTTCCAGACCAAC**SNN**ACGAACAATACCATTTGCAC
D255_NNS_fwd<br>D255_NNS_rev	GACAGGTCAGTTTTTT**NNS**TTTAGCGGTGGTTGGGG<br>CCCCAACCACCGCTAAA**SNN**AAAAAACTGACCTGTC
D65_NNS_fwd<br>D65_NNS_rev	CTGGCAAAACGTGGTGCA**NNS**GTTCTGACCATTAGTGATG<br>CATCACTAATGGTCAGAAC**SNN**TGCACCACGTTTTGCCAG
R226_NNS_fwd<br>R226_NNS_rev	GCACAGGTTCCGCTG**NNS**CGTCTGGGTACAATG<br>CATTGTACCCAGACG**SNN**CAGCGGAACCTGTGC
T123W_fwd<br>T123W_rev	CAGTGGGCAAAAGTTAAAGCA**TGG**AACCTGGATATGGTTTTTC<br>GAAAAACCATATCCAGGTT**CCA**TGCTTTAACTTTTGCCCACTG
A144S_fwd<br>A144S_rev	CTCCGATGGTTGCAGCCGGT**AGC**GGTCAGTGTGTTGTTTTTAC<br>GTAAAAACAACACACTGACC**GCT**ACCGGCTGCAACCATCGGAG
M161G_fwd<br>M161G_rev	TGGTCGTCCGGATCCG**GGC**GTGAGCATTTATGGTG<br>CACCATAAATGCTCACGC**CCG**GATCCGGACGACCA
C147_NNS_fwd<br>C147_NNS_rev	CAGCCGGTGCAGGTCAG**NNS**GTTGTTTTTACCAGCGC<br>GCGCTGGTAAAAACAAC**SNN**CTGACCTGCACCGGCTG
T123_NNS_fwd<br>T123_NNS_rev	GTGGGCAAAAGTTAAAGCA**NNS**AACCTGGATATGGTTTTTC<br>GAAAAACCATATCCAGGTT**SNN**TGCTTTAACTTTTGCCCAC
G174M_fwd<br>G174M_rev	CCCGTGCCGGTGCAAAT**ATG**ATTGTTCGTGCAGTTG<br>CAACTGCACGAACAAT**CAT**ATTTGCACCGGCACGGG
V46P_Fwd<br>V46P_rev	GCCGGTGATGGCACCATG**CCG**GGTGTTGAAGAAAGTTTTG<br>CAAAACTTTCTTCAACACC**CGG**CATGGTGCCATCACCGGC
C147I_fwd<br>C147I_rev	GCCGGTGCAGGTCAG**AT**TGTTGTTTTTACCAGC<br>GCTGGTAAAAACAACA**AT**CTGACCTGCACCGGC
R208G_fwd<br>R208G_rev	GTTTTCTGAAAGCAAGC**GGC**GCAGATGGTGATCCGG<br>CCGGATCACCATCTGC**GCC**GCTTGCTTTCAGAAAAC

Protein expression and purification in shake flasks

For heterologous production of site-directed variants of HheG in *E. coli* BL21 (DE3) Gold, 20 or 60 mL TB medium supplemented with 50 mg L^{-1} kanamycin was inoculated and incubated overnight at 37 °C and 200 rpm. 10 or 50 mL of this pre-culture were added to 100 or 500 mL TB medium, respectively, supplemented with 50 mg L^{-1} kanamycin and 0.2 mM IPTG as inducer. Positive hits from the library screening and point mutants were produced in 100 mL scale for validation of apparent melting temperatures and determination of specific activities after purification, whereas HheG WT and mutants T123W, T123Y, T123H, T123F and T123G were additionally produced in 500 mL scale for detailed enzyme characterization. Expression was performed at 22 °C and 200 rpm for 24 h. Cells were harvested by centrifugation (3488 g, 20 min, 4 °C), cell pellets were washed once with 50 mM Tris/SO_4 buffer, pH 7, centrifuged again and stored at -20°C until further use.

Protein purification from cell pellets of 100 mL expression cultures was performed using gravity flow columns containing 2 mL Ni Sepharose 6 Fast Flow resin (GE Healthcare Life Sciences, Freiburg, Germany), which were equilibrated with 5 column volumes of binding buffer (50 mM Tris/SO_4, 300 mM Na_2SO_4, 25 mM imidazole, pH 7.9). First, cell pellets were re-suspended in 10 mL lysis buffer and cells were disrupted by sonication (5 min, 65% amplitude, cycles of 10 s pulse and 20 s pause). Cell debris was removed by centrifugation (18533 g, 45 min, 4 °C) and after filtration through a 0.45 µm cellulose acetate membrane filter (Sarstedt, Nümbrecht, Germany), the resulting CFE was loaded on the column. Then, the column was washed with 3 column volumes of binding buffer before elution of the His-tagged protein with 5 column volumes of elution buffer (50 mM Tris/SO_4, 300 mM Na_2SO_4, 400 mM imidazole, pH 7.9). Protein-containing fractions were identified using the Bradford assay[9].

Purification of the respective His-tagged enzymes from 500 mL expressions was performed on an Äkta pure FPLC (GE Healthcare Life Sciences, Freiburg, Germany) according to the protocol of Koopmeiners et al.[10] with the following modifications: His-tagged enzyme was eluted using a 60 mL gradient from 25 to 500 mM imidazole with a flow rate of 1 mL min^{-1}.

Activity and stereoselectivity determination

Activities of positive hits from the Thermofluor-based library screening were analyzed in bioconversions of 1.4 mL total volume containing 20 mM cyclohexene oxide (from a 1 M stock in DMSO), 40 mM azide and 100 µg enzyme in 50 mM Tris/SO_4 buffer, pH 7 at 22 °C. 400 µL samples were taken after 30 min of reaction and an equal amount of *tert*-butyl methyl ether (TBME) supplemented with 0.1% (v/v) dodecane as internal standard was used for extraction.

After drying the organic phase over anhydrous $MgSO_4$, samples were analyzed on achiral GC. Negative control reactions without enzyme as well as reactions with HheG WT were carried out in parallel. Specific activities were calculated based on the amount of formed product after 30 min reaction time.

Identical reaction conditions were also used in conversions with HheG wild type and the T123 mutants for the determination of product enantiomeric excesses. For this, samples were analyzed on chiral GC.

Initial reaction velocities of T123 mutants and HheG wild-type were determined in reactions containing 20 mM cyclohexene oxide (from a 1 M stock in DMSO), 40 mM azide and 50 µg enzyme in 50 mM Tris/SO_4 buffer at pH 7 in a total volume of 1.6 mL. Reactions were incubated at 22 °C. 400 µL samples were taken after 5, 10, 15 min and extracted using an equal amount of TBME supplemented with 0.1% (v/v) dodecane. Samples were analyzed on achiral GC. Negative control reactions without enzyme were carried out in parallel.

Initial reaction velocities of HheG wild-type and mutants T123W and T123G were also determined in reactions containing 20 mM (+)-limonene oxide, (2*R*,3*R*)- or (2*S*,3*S*)-2-methyl-3-phenyloxirane (all from 1 M stocks in DMSO), 40 mM azide and 20 µg enzyme (in case of (+)-limonene oxide) or 100 µg enzyme (in case of 2-methyl-3-phenyloxirane) in 50 mM Tris/SO_4 buffer at pH 7 in a total volume of 1.5 mL. Reactions were incubated at 22 °C. 300 µL samples were taken after 3, 5.5, 8 min and extracted using an equal amount of TBME supplemented with 0.1% (v/v) dodecane. Samples were analyzed on achiral GC. Negative control reactions without enzyme were carried out in parallel.

To determine the effect of different co-solvents on initial reaction velocities of HheG wild-type and mutants T123W and T123G, 1.6 mL reactions were carried out as described above but with substrate from 1 M stock solutions prepared in different organic solvents (dimethylsulfoxide, ethanol, methanol, dimethylformamide, acetonitrile or isopropyl alcohol). To obtain 5, 10 and 25% final co-solvent concentrations in the reactions, an extra amount of respective co-solvent was added. Again, 400 µL samples were taken after 5, 10, 15 min and extracted as described above for subsequent achiral GC analysis.

Temperature profiles

Temperature profiles of wild-type HheG as well as variants T123W and T123G were determined as previously described[10], but using 0.1 mg mL^{-1} purified enzyme.

CD measurements

Circular dichroism measurements were performed for wild-type and variants T123W, T123F and T123G on a Jasco J-715 spectropolarimeter in a UV quartz cuvette with a diameter of 0.1 mm combined with a Huber Polystat cc2. The measured spectrum range was 185 nm – 260 nm and the protein concentrations varied between 1.5 and 2 mg mL^{-1} (in TE buffer). Spectra for each protein were measured at different temperatures and the ellipticity minimum of each curve was plotted against the temperature. The local maximum of the first derivative of the fitted Boltzmann curve yielded the apparent melting temperature (T_m).

Analytical methods

Achiral and chiral GC analyses were performed on a GC2010 plus gas chromatograph (Shimadzu, Duisburg, Germany) with FID detection. Achiral separation was carried out using an OPTIMA 5 MS column (Macherey Nagel, Düren, Germany) with a length of 30 m, inner diameter of 0.25 mm and a film thickness of 0.25 µm. Separation of cyclohexene oxide and product was achieved using a temperature program of 110 °C, 7.5 min//50 °C min^{-1} //295 °C, 2.0 min with a total flow of 1.24 mL min^{-1} and hydrogen as carrier gas. Substrate and product eluted at retention times of 2.0 min and 5.4 min, respectively. Separation of (+)-limonene oxide, (2*R*, 3*R*)-2-methyl-3-phenyloxirane and (2*S*, 3*S*)-2-methyl-3-phenyloxirane as well as respective products was achieved using a temperature program of 80 °C, 1 min//10 °C min^{-1}//160 °C//20 °C min^{-1}//300 °C with a total flow of 1 mL min^{-1} and hydrogen as carrier gas. The retention times of the substrates and produts were: 6.7 min (2-methyl-3-phenyloxirane), 7.0 min ((+)-*cis*-limonene oxide), 7.1 min ((+)-*trans*-limonene oxide), 10.3 min (1-azido-1-phenylpropan-2-ol), 10.4 min (2-azido-1-phenylpropan-1-ol), 10.9 min ((+)-*cis*-2-azido-1-methyl-4-(prop- 1-en-2-yl)cylohexan-1-ol) and 11.3 min ((+)-*trans*-2-azido-1-methyl-4-(prop-1-en-2-yl)cylohexan-1-ol).

Chiral separation of azidocyclohexanol enantiomers was carried out using a HYDRODEX γ-DIMOM column (Macherey-Nagel, Düren, Germany) with a length of 25 m, inner diameter of 0.25 mm and a film thickness of 0.25 µm. Separation was achieved using a temperature program of 100°C, 40 min//10 °C min^{-1} //200 °C, 2 min with a total flow of 1.2 mL min^{-1} and hydrogen as carrier gas. Retention times were as follows: cyclohexene oxide (3.3 min), (1*S*, 2*S*)-2-azidocyclohexan-1-ol (37.1 min) and (1*R*, 2*R*)-2-azidocyclohexan-1-ol (38.5 min).

References

1. Chen, J., Lu, Z., Sakon, J. & Stites, W. E. Increasing the thermostability of staphylococcal nuclease: Implications for the origin of protein thermostability. *J. Mol. Biol.* **303,** 125–130 (2000).

2. Reetz, M. T., Soni, P., Fernández, L., Gumulya, Y. & Carballeira, J. D. Increasing the stability of an enzyme toward hostile organic solvents by directed evolution based on iterative saturation mutagenesis using the B-FIT method. *Chem. Commun.* **46,** 8657–8658 (2010).

3. Floor, R. J. *et al.* Computational library design for increasing haloalkane dehalogenase stability. *ChemBioChem* **15,** 1660–1672 (2014).

4. Koudelakova, T. *et al.* Engineering enzyme stability and resistance to an organic cosolvent by modification of residues in the access tunnel. *Angew. Chemie - Int. Ed.* **52,** 1959–1963 (2013).

5. Khan, A. R., Nirasawa, S., Kaneko, S., Shimonishi, T. & Hayashi, K. Characterization of a solvent resistant and thermostable aminopeptidase from the hyperthermophillic bacterium, *Aquifex aeolicus*. *Enzyme Microb. Technol.* **27,** 83–88 (2000).

6. Hao, J. & Berry, A. A thermostable variant of fructose bisphosphate aldolase constructed by directed evolution also shows increased stability in organic solvents. *Protein Eng. Des. Sel.* **17,** 689–697 (2004).

7. Di, L. & Kerns, E. H. Biological assay challenges from compound solubility: strategies for bioassay optimization. *Drug Discov. Today* **11,** 446–451 (2006).

8. Arabnejad, H. *et al.* A robust cosolvent-compatible halohydrin dehalogenase by computational library design. *Protein Eng. Des. Sel.* **30,** 175–189 (2017).

9. Bradford, M. M. A rapid and sensitive method for the quantitation of microgram quantities of protein utilizing the principle of protein-dye binding. *Anal. Biochem.* **72,** 248–254 (1976).

10. Koopmeiners, J. *et al.* HheG, a halohydrin dehalogenase with activity on cyclic epoxides. *ACS Catal.* **7,** 6877–6886 (2017).

11. Bednar, D. *et al.* FireProt: Energy- and evolution-based computational design of thermostable multiple-point mutants. *PLoS Comput. Biol.* **11**, e1004556;

10.1371/journal.pcbi.1004556 (2015).

12. Adams, P. D. *et al. PHENIX*: A comprehensive Python-based system for macromolecular structure solution. *Acta Crystallogr. Sect. D Biol. Crystallogr.* **66,** 213–221 (2010).
